# Proceedings of a Convention of the Physicians of Kentucky

**Published:** 1851-11

**Authors:** D. W. Yandell

**Affiliations:** Louisville, Ky.


*Proceedings of the State Medical Convention.*—We are indebted to the polite attention of Doctor D. W. YANDELL, for a copy of the proceedings of the medical convention recently assembled at Frankfort, Ky. It is earnestly to be hoped, that the medical profession of Kentucky will now awaken to the magnitude of the interests connected with a State Medical Society, and secure the full success of the present enterprize. County and district medical societies should be established all over the State, and the entire regular profession should be enrolled in regularly organised bodies. In almost every State of the Union, the members of the medical profession are engaged in efforts to improve the condition of the profession, and the proceedings of most of these State societies bear strong testimony to the usefulness of such institutions. When her sister States are advancing in useful works, Kentucky should not be a sluggard, and surely there is enough public spirit, honorable emulation, and professional pride among the medical men of Kentucky to enable them to do their duty towards the progress of the profession. But we must not rest upon our oars now, under the impression that all that is needful has been done, because a medical convention has met at Frankfort, and adopted a constitution for a State Medical Society. Such things as these have been done before, without any very marvellous results, and it behooves each physician who feels an interest in the cause to go to work, and, by activity, zeal according to knowledge, and perseverance to secure success in the effort to organise the medical mind in Kentucky.

Let those who have the cause of medical progress at heart, who know the value of medical science, and the usefulness of fraternising in medical societies in furthering these objects remember, that each member of the profession has a duty to perform. If any one is zealous, active and energetic in the cause, let him impress a portion of these virtues upon as many of his brethren as he can reach. It must not be forgotten that in 1841, a medical convention assembled in Frankfort, and adopted measures eminently calculated to elevate and enoble the whole profession. The proceedings were harmonious, and everything that was done by the convention was characterised by a disposition to benefit each individual physician, and by this means to advance the general good of all the members. An excellent constitution for a State Medical Society was adopted; a code of medical ethics was incorporated into the constitution, and there was reason to hope from the whole of the proceedings, that the medical profession in Kentucky was awaking to its true interests. But the good and the benefits ended with the initial steps taken by the convention. The labors of that body were quietly permitted to die, and nothing more was heard of them. We refer to these matters of the past in order to show those who honestly desire professional advancement that they must work for it. These things do not fall into the lap of indolence; those who desire the good of their profession must assiduously work for it.

We have every reason to hope for benefit from the labors of the recent medical convention at Frankfort, and and those hopes may end in full fruition, provided physicians throughout the State shall second the efforts of the convention, and come forward to aid and encourage the cause. We earnestly urge these matters on the attention of the profession in Kentucky, and beg of its members their hearty co-operation with those who have come forward in the cause of medical advancement.—*Ed. Western Journal Med. & Surg.*

ART. II.—*Proceedings of a Convention of the Physicians of Kentucky.*

At a convention of the physicians of Kentucky, held in the Senate Chamber at Frankfort, on the 1st day of October, 1851, at 10 o'clock, A. M., Dr. W. L. SUTTON, of Georgetown, was called to the Chair, and Drs. E. H. WATSON and J. M. MILLS, of Frankfort, appointed Secretaries.

The meeting was opened with prayer by Rev. Dr. Robinson.

On motion of Dr. Chipley, a committee consisting of Drs. Evans, Chipley and Breckenridge were appointed to ascertain the names and localities of the physicians in attendance, who reported the following:

Drs. S. D. Gross, Henry Miller, W. H. Miller, D. W. Yandell, T. G. Richardson, D. D. Thomson, R. J. Breckinridge, Jr., N. B. Anderson and J. B. Flint, of Louisville; Dr. E. D. Foree, of Jefferson county; Drs. J. Dudley and J. P. Letcher, of Nicholasville; Drs. W. L. Sutton, Henry Craig and J. D. Winston, of Georgetown; Drs. W. C. Sneed, H. Rodman, C. G. Phythian, E. H. Watson, Ben. Monroe, Jr., J. G. Roberts, J. M. Mills, and Ben. Hensley, of Frankfort; Dr. L. Y. Hodges, Franklin county; Dr. R. W. Evans, Mercer county; Dr. A. Evans, Covington; Dr. W. R. Chew, Woodford county; Dr. C. H. Spillman, Harrodsburg, Dr. Geo. B. Harrison, Fayette county; Drs. W. S. Chipley, and D. J. Ayres, Lexington; Drs. L. G. Ray, and Edward Ingles, Paris; Drs. E. H. Black, and Jas. Adams, Scott county; Drs. D. S. Slaughter, and R. W. Glass, Shelbyville; Dr. Joshua Gore, Nelson county; and Dr. E. C. Drane, Henry county.

Dr. Flint offered the following resolution:

*Resolved*, That ——————————————— be a committee to report to this convention, a suitable address to the profession of the State, calling upon them to assemble at such time and place as this meeting may advise, for the purpose of organising a permanent State Medical Society, and that in the meantime, we take steps at once to connect the profession of our State with the national organization, by appointing delegates to the next annual meeting of the American Medical Association.

Dr. Breckinridge moved the following as a substitute for the resolution of Dr. Flint; strike out all after the word "resolved," and insert: "That a committee be appointed to report the order of business for the convention now assembled."

The substitute and original resolution were laid on the table, and a committee consisting of Drs. Chipley, A. Evans, Spillman, Dudley and Sneed, were appointed to draft a constitution for the formation of a State Medical Society.

The convention adjourned until 2½ o'clock, P. .M.

2½ O'CLOCK, P. M.

The convention was called to order by the president, Dr. Chipley, from the committee appointed to draft a constitution, presented the following report, which, on motion of Dr. Gross, was received.*

The constitution was taken up, each article especially considered, and after some amendments was adopted as a whole.

On motion of Dr. Gross, a committee consisting of one from each city and county now represented, entitled to a representative in the state Legislature, be appointed to nominate officers for the present year. The following

*The constitution will be printed and circulated in pamphlet form.

gentlemen were appointed: Drs. Breckinridge, of Louisville; Foree, of Jefferson; Letcher, of Jessamine; Spillman, of Mercer; Harrison, of Fayette; Roberts, of Franklin; Gore, of Nelson; Slaughter, of Shelby; Evans, of Kenton; Chew, of Woodford; and Black, of Scott; who after a short interval, reported the following nominations:

For President, Dr. W. L. Sutton, of Georgetown;
" Sen. Vice President, Dr. W. S. Chipley, of Lexington;
" Jun. " " Dr. J. Dudley, of Nicholasville;
" Recording Secretary, Dr. W. C. Sneed, of Frankfort;
" Corresponding " Dr. R. J. Breckinridge, Jr. Louisville;
" Treasurer, Dr. R. W. Glass, of Shelbyville;
" Librarian, Dr. Ben. Monroe, of Frankfort.

The report was received, and on balloting for each officer separately, the gentlemen nominated by the committee were declared duly elected.

Drs. J. M. Mills, E. H. Watson, and W. C. Sneed were elected a committee of publication.

The convention then adjourned *sine die.*

---

*Proceedings of the Kentucky State Medical Society.*—At a meeting of the State Medical Society of Kentucky, held in the capitol at Frankfort, on the 1st day of October, 1851, at 5 o'clock, P. M., the President Dr. Sutton took the chair, and called the society to order.

On motion of Dr. Ayres, a committee consisting of Drs. Dudley, Yandell, Harrison, Roberts, and Winston, were appointed to apply to the ensuing session of the Legislature of Kentucky for a charter for this body.

On motion of Dr. Chipley, the next annual meeting of this society was ordered to be held in the city of Louisville, on the third Wednesday in October, 1852.

Dr. Richardson presented the following resolution, which was adopted:

*Resolved*, That the Code of Medical Ethics of the American Medical Association be adopted by this society.

Dr. Chipley presented a form of charter for the society, which after some discussion was withdrawn.

On motion of Dr. Breckinridge, a committee consisting of Drs. Rodman, Anderson, Thomson, Ayres, and Spillman were appointed to draft and report a set of by-laws.

The application of Dr. J. C. Darby, of Lexington, was presented for membership, and the society then adjourned until 7½ o'clock.

7½ O'CLOCK, P. M.

The society was called to order by the President.

The application of Dr. J. C. Darby was taken up, and that gentleman was duly elected a member of the society.

The President then announced the following chairmen of the various standing committees:

| | | |
|---|---|---|
| Chairman Com. Arrangements, | Dr. Anderson, | Louisville; |
| " " Medical Ethics, | Dr. A. Evans, | Covington; |
| " " Public Hygiene, | Dr. Flint, | Louisville; |
| " " Vital Statistics, | Dr. Chipley, | Lexington; |
| " " Epidemics. | Dr. Darby, | " |
| " " Obstetrics, | Dr. H. Miller, | Louisville; |
| " " Imp. in Prac. Med., | Dr. Foree, | Jefferson co; |
| " " " " in Surgery, | Dr. Gross, | Louisville; |
| " " " " in Pharmacy, | Dr. Mills, | Frankfort; |
| " Com. Indiginous Botany, | Dr. Spillman, | Harrodsburg; |
| " " Finance, | Dr. Thomson, | Louisville. |

Dr. Flint declined serving as chairman of the committee on Hygiene. Dr. Drane was appointed to fill the vacancy.

On motion of Dr. Breckinridge, the President was appointed chairman of a committee to memorialize the Legislature upon the subject of registration of marriages, births and deaths.

The committee on by-laws were granted until the next regular meeting to report.

On motion of Dr. Breckinridge, the society determined to go into the election of Honorary members—the vote was then reconsidered, and the subject for the present postponed.

The society elected the following persons as delegates, to the American Medical Association, viz: Drs. L. G. Ray, E. D. Foree, T. G. Richardson, D. J. Ayres, D. S. Slaughter, E. C. Drane, W. H. Miller, W. R. Evans, and Joshua Gore.

The President presented a series of resolutions, which were laid over until the next regular meeting.

On motion of Dr. Gross, the President of the Society was requested to deliver an opening address at the next annual meeting.

The record of proceedings was read, and after slight amendments was adopted, and ordered to be published in connection with the constitution.

A vote of thanks was tendered to the officers of the society for their prompt and efficient discharge of duty; and then the society adjourned. W. L. SUTTON, *Pres.*

W. C. SNEED, *Sec'y.*

